# Electrochemical Genotoxicity Assay Based on a SOS/*umu* Test Using Hydrodynamic Voltammetry in a Droplet

**DOI:** 10.3390/s121217414

**Published:** 2012-12-14

**Authors:** Hideki Kuramitz, Kazuto Sazawa, Yasuaki Nanayama, Noriko Hata, Shigeru Taguchi, Kazuharu Sugawara, Masami Fukushima

**Affiliations:** 1 Department of Environmental Biology and Chemistry, Graduate School of Science and Engineering for Research, University of Toyama, Gofuku 3190, Toyama 930-8555, Japan; E-Mails: d1071303@ems.u-toyama.ac.jp (K.S.); seven-mountain@kde.biglobe.ne.jp (Y.N.); noriko@sci.u-toyama.ac.jp (N.H.); taguchi@sci.u-toyama.ac.jp (S.T.); 2 Faculty of Engineering, Maebashi Institute of Technology, Maebashi, Gunma 371-0816, Japan; E-Mail: kzsuga@maebashi-it.ac.jp; 3 Laboratory of Chemical Resources, Division of Sustainable Resources Engineering, Graduate School of Engineering, Hokkaido University, Sapporo 060-8628, Japan; E-Mail: m-fukush@eng.hokudai.ac.jp

**Keywords:** SOS/*umu* genotoxicity test, hydrodynamic voltammetry, rotating disk electrode, microdroplet sample, humic acid, sediment

## Abstract

The SOS/*umu* genotoxicity assay evaluates the primary DNA damage caused by chemicals from the β-galactosidase activity of *S. typhimurium*. One of the weaknesses of the common *umu* test system based on spectrophotometric detection is that it is unable to measure samples containing a high concentration of colored dissolved organic matters, sediment, and suspended solids. However, *umu* tests with electrochemical detection techniques prove to be a better strategy because it causes less interference, enables the analysis of turbid samples and allows detection even in small volumes without loss of sensitivity. Based on this understanding, we aim to develop a new *umu* test system with hydrodynamic chronoamperometry using a rotating disk electrode (RDE) in a microliter droplet. PAPG when used as a substrate is not electroactive at the potential at which PAP is oxidized to *p*-quinone imine (PQI), so the current response of chronoamperometry resulting from the oxidation of PAP to PQI is directly proportional to the enzymatic activity of *S. typhimurium*. This was achieved by performing genotoxicity tests for 2-(2-furyl)-3-(5-nitro-2-furyl)-acrylamide (AF-2) and 2-aminoanthracene (2-AA) as model genotoxic compounds. The results obtained in this study indicated that the signal detection in the genotoxicity assay based on hydrodynamic voltammetry was less influenced by the presence of colored components and sediment particles in the samples when compared to the usual colorimetric signal detection. The influence caused by the presence of humic acids (HAs) and artificial sediment on the genotoxic property of selected model compounds such as 4-nitroquinoline-*N*-oxide (4-NQO), 3-chloro-4-(dichloromethyl)-5-hydroxy-2(5*H*)-furanone (MX), 1,8-dinitropyrene (1,8-DNP) and 1-nitropyrene (1-NP) were also investigated. The results showed that the genotoxicity of 1-NP and MX changed in the presence of 10 mg·L^−1^ HAs. The genotoxicity of tested chemicals with a high hydrophobicity such as 1,8-DNP and 1-NP were decreased substantially with the presence of 1 g·L^−1^ sediment. This was not observed in the case of genotoxins with a low log *K*_ow_ value.

## 1. Introduction

The advancement of industrialization, agricultural and urban activities have caused serious concerns about chemical substances that are discharged to the air, soil and water environment. They have a direct impact on the ecosystem and pose a threat to human health. The transformations of chemicals which accumulate in the environment can produce secondary pollutants from reactions with other chemicals. The absolute concentrations of known analytes in the environment can be determined by chemical analysis methods. However, these kinds of techniques do not provide adequate interpretation of their toxicities to biota. In order to gain a better understanding of the risk posed by various pollutants, there is an inevitable need for an effective analytical approach which is able to access the biological impacts. The use of bioassays in analytical research has provided an accurate evaluation of environmental risk.

Many researchers have reported on the existence of different mutagenic and carcinogenic compounds in river waters, suspended solids, soils, sediments and treated drinking waters [1–13]. These findings indicate a high concern about genotoxins in aquatic biota and their possible effects on human health. Microorganisms, bacteria in particular, have several attributes which make them attractive to be used in mutagenicity assays. Generally, microbial tests are simpler and more inexpensive than methods based on tissue culture and animal experiments. The *Salmonella*/microsome test (Ames test) and SOS induction assay (*umu* test) are two of the most common genotoxicity assays. The Ames test is based on a set of *Salmonella typhimurium* strains that revert to histidine independence upon exposure to mutagens [14]. On the other hand, *umu* test is one of the more rapid screening systems for genotoxins. This system is based on the ability of a number of chemicals to induce *umu* gene expression in a new tester strain *S. typhimurium* TA1535/pSKI002 in which an *umuC‘-’lacZ* fused gene had been introduced. Thus, the potentials of chemicals to induce SOS response can be monitored by measuring the cellular β-galactosidase activity produced by fusion gene [15–17]. The β-galactosidase activity is usually determined by measuring the products of the enzymatic reaction using UV/vis spectrometry or fluorometry. Reifferscheid *et al*. reported that the *umu* test was able to detect 86% of the Ames mutagens, while the Ames test (using at least five strains) detected 97% of the *umu* positive compounds [18]. The *umu* test is useful as a mass screening method because it can be carried out in a common 96-well microplate [19]. However, the *umu* test cannot be applied in direct evaluation of samples containing colored dissolved organic matter, sediment, and high concentrations of suspended matter because it depends on the spectrophotometric detection. Thus, the adsorbed genotoxins in the soil, sediment and suspended solids are assessed after extracting by an organic solvent such as hexane, dichloromethane, and acetone [1,3,5,10]. This extraction process not only complicates the assay operation but it is also makes impossible the evaluation of the genotoxicity changes caused by the presence of organic matter, suspended solids, soil and clay particles, *etc*.

Humic substances (HSs) are heterogeneous mixtures of polydispersed materials formed by biochemical and chemical reactions during the decay and transformation of plant and microbial matter that remains in soils, sediments and natural water. HSs are the most widely distributed natural organic compounds in various environments. The dissolved humic substances (DHSs) known as fulvic acid (FA) and humic acid (HA) are the main components of dissolved organic matter (DOM) in water [20]. HSs have affinity for a variety of heavy metal ions and hydrophobic organic compounds. Previous studies have reported on the adsorption behaviors of pollutants to HSs and soil particles [21–23]. Some of these studies even focused on the relationships between the partition coefficient (*K*_oc_) of organic pollutants and the chemical characteristics of DHSs [24–29]. HSs play an important role in aquatic and soil environment because they are strongly related to the changes in speciation and toxicity of numerous pollutants. Previous studies have showed that DHSs reduce the toxicity of pollutants such as cadmium, zinc, copper and DDT, *etc*. to zooplankton [30–33] and fishes [34,35]. However, reports on the changes in genotoxicity of pollutants to microbial species and animals caused by the presence of natural organic matters and soil particles are still scarce [36,37].

There are many advantages of eletrochemical detection in bioassays. For example, it is relatively a simple, rapid, inexpensive, sensitive and user-friendly method. It is applicable to optically opaque media and allows detection of a small sample volume without loss of sensitivity, unlike optical detection techniques such as UV/vis spectrometry and fluorometric detection. In some recent works, the benefits of electrochemical detection have been employed into genotoxicity assays based on the *umu* test [38,39]. For these assays, *p*-aminophenyl-β-D-galactopyranoside (PAPG) is used as a substrate to evaluate the enzymatic reaction. The β-galactosidase activity of *S. typhimurium* is determined by measuring the electrochemical oxidation of *p*-aminophenol (PAP) which is converted from PAPG by β-galactosidase.

**Figure 1.**
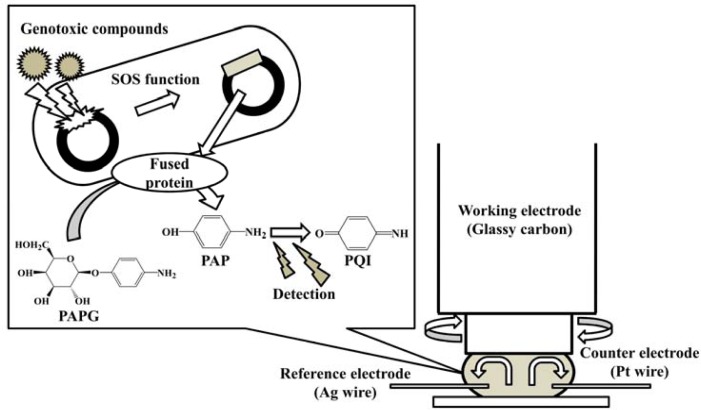
Schematic diagram of the *umu* test with hydrodynamic electrochemical detection using a rotating disk electrode in a microdroplet.

The purpose of this study is to develop a new electrochemical genotoxicity test which enables the analysis of turbid samples. It is based on the *umu* test with hydrodynamic electrochemical detection using a rotating disk electrode (RDE) in a microliter (*ca*. 45 μL) droplet, as shown in Figure 1. The RDE has a dual function. It can be used to effectively mix the sample solution and at the same time acts as an electrochemical detection device. The fast rotation speed of the RDE promotes the convective mass transport to the electrode surface. This decreases the incubation time for the enzymatic reaction. The advantages of using a microdroplet as a reaction vessel are reduced volume of reagents and waste produced, and as well as lower detection limits with shorter assay times [40–42]. The hydrodynamic electrochemical *umu* test proposed in this study was demonstrated with samples containing high concentration of DHSs and sediment particles, and it was compared with the regulatory *umu* test based on UV/vis spectrometric detection. Genotoxicity changes for several mutagens (4-nitroquinoline-*N*-oxide (4-NQO), 3-chloro-4-(dichloromethyl)-5-hydroxy-2(5*H*) furanone (MX), 1,8-dinitropyrene (1,8-DNP) and 1-nitropyrene (1-NP) caused by the presence of HAs from different sources and artificial sediment (*Sphagnum* moss peat 4%, kaolin 20%, quartz sand 76%, particle size is ≤25 μm) in the sample were evaluated.

## 2. Experimental Section

### 2.1. Chemicals

Bacto tryptone was obtained from Difco Co. (Detroit, MI, USA). Ampicillin, *o*-nitrophenyl-β-D-galactopyranoside (ONPG) and *p*-aminophenyl-β-D-galactopyranoside (PAPG) were from Sigma-Aldrich (St. Louis, MO, USA). D-(+)-Glucose, dimethyl sulfoxide (DMSO), 2-(2-furyl)-3-(5-nitro-2-furyl)-acrylamide (AF-2, log *K*_ow_ = 1.14), 2-aminoanthracene (2-AA, log *K*_ow_ = 4.13), 4-nitro-quinoline-*N*-oxide (4-NQO, log *K*_ow_ = 0.82), 3-chloro-4-(dichloromethyl)-5-hydroxy-2(5*H*)-furanone (MX, log *K*_ow_ = 0.61), 1,8-dinitropyrene (1,8-DNP, log *K*_ow_ = 4.57), 1-nitropyrene (1-NP, log *K*_ow_ = 4.75), kaolin and quartz sand were from Wako Pure Chemical Industry Ltd. (Osaka, Japan). The values for log*K*_ow_ of genetoxins were calculated by using KOWWIN v 1.68. Liver 9,000 × *g* supernatant fractions (S9) were prepared from rats pretreated with phenobarbital and 5,6-benzoflavone. The cofactors were obtained from Oriental Yeast Co. (Tokyo, Japan). The *Sphagnum* moss peat was purchased from a local market in Toyama, Japan. Kaolin, quartz sand, ground into a fine powder using a mortar and pestle, and passed through a 25 μm mesh sieve. All reagents used were of analytical grade, and water was sterile deionized water. The phosphate buffer solution (PBS, pH 7.4) consisted of 0.044 M NaH_2_PO_4_, 0.056 M Na_2_HPO_4_. B-buffer (pH 7.0) consisted of 0.1 M PBS, 0.01 M potassium chloride, 0.5 mM magnesium sulfate, 0.1% sodium dodecyl sulfate (SDS) and 0.05 M mercaptoethanol.

### 2.2. Bacterial Strain

Salmonella typhimurium NM2009 (TA1535/pSK1002/pNM12) was used as a bacterial strain. The strain was provided by Protein Purify Ltd. (Gunma, Japan). Tester strain NM2009 was constructed by subcloning the bacterial *O*-acetyltransferase (*O*-AT) gene into a plasmid vector pACYC184 and introducing the plasmid into the original strain *S. typhimurium* TA1535/pSK1002 harboring an *umuC‘-’lacZ* fusion gene [43–46]. *O*-AT is suggested to catalyze key steps in the mutagenic activation of aromatic amines. NM2009 strain indicated high mutagenic responses by aromatic amines [46].

### 2.3. Isolation and Purification for Humic Acids

Four different kinds of HAs were used as DHSs. A commercially available HA was obtained from Wako Pure Chemical Co. (WHA). This was dissolved in 0.1 M NaOH, and the solution was then treated with mixture of HF and HCl. The resulting precipitate was transferred to a dialysis tube (molecular weight cutoff 500 Da). After dialysis, the slurry was freeze dried. The soil, peat and sediment samples used in the isolated HAs were collected from tropical peat land (Central Kalimantan, Indonesia, KPHA), peat land of Shinshinotsu town (Hokkaido, Japan, SPHA), high moor peat of Amou moor (Gifu, Japan, APHA), brown forest soil (Toyama, Japan, FSHA). HAs of soil, peat and sediment were extracted by the method based on the International Humic Substances Society (IHSS) [47]. Prior to use the experiments, the purified HA was dissolved in 1 M NaOH solution (>pH 10) and stirred for 30 min. The pH of solution was adjusted to pH 8.0 with 1 M HCl.

### 2.4. Characterization of HAs

HAs used in this study were characterized by means of UV/vis spectroscopy, functional group analysis, elemental analysis, and size exclusion chromatography. The results for characterization of HAs are shown in Table 1.

#### 2.4.1. Spectroscopic Characterization

The absorbance values at 250, 280, 365, 400, and 600 nm were recorded for the calculation of *E*4/*E*6, *E*280, *E*2/*E*3. The absorptivities at 280 nm and 400 nm were calculated as: *E* (1 cm^−1^·g^−1^ of C) = absorbance/[HS (g·L^−1^)] × (% C/100). The quotient *E*4/*E*6 (the ratio of absorbance at 400 and 600 nm) is widely used in soil science as an indicator for humification [48]. In generally, the progressive humification is indicated by decreasing of *E*4/*E*6 ratios. *E*280 and *E*2/*E*3 (the ratio of absorbance at 250 and 365 nm) correlated strongly with the total aromaticity and averaged molecular weight of all humic solutes [48].

#### 2.4.2. Functional Group Analysis

The amount of total acidity and carboxylic acid content were measured by potentiometric methods using the barium hydroxide and Ca-acetate methods, respectively [20]. The contents of phenolic hydroxyl groups were calculated by subtracting the carboxylic acid contents from the total acidity.

#### 2.4.3. Elemental Analysis

The analysis of C, H, N, S and ash contents of HAs were carried out by means of a Micro Corder JM 10 type CHN analyzer (J-Science Lab. Co. Ltd., Kyoto, Japan). The sulfate ion, which is absorbed in H_2_O_2_ aqueous, was analyzed using a DX-500 type ion chromatography (Dionex, Sunnyvale, CA, USA). The percentage of oxygen is found by subtracting the total sum percentage of C, H, N, S, and ash elements from 100.

#### 2.4.4. Estimation of Molecular Weight by Size Exclusion Chromatography

A three hundred μL aliquot of 1 g·L^−1^ HAs solution dissolved in 0.01 M NaOH and 900 μL of 0.01 M phosphate buffer (pH 7.0) and acetonitrile (75/25 = v/v) was mixed. To determine the molecular weight of HAs, a 20 μL aliquot was injected into a Jasco PU-2080 plus Intelligent HPLC pump system equipped with a UV-2075 UV/vis detector (Japan Spectroscopic Co., Tokyo, Japan). The mobile phase consisted of a mixture of 0.01 M phosphate buffer (pH 7.0) and acetonitrile (75/25 = v/v), and the flow rate was set at 0.75 mL·min^−1^. A TSK-Gel α-M column (Tosoh, 7.8 mm inner diameter × 300 mm) was used as the solid phase, and the column temperature was maintained at 40 °C. The UV absorption of HAs was measured at 260 nm. Reference substances for construction of molecular weight calibration curves were sodium salts of polystyrene sulfonates with molecular weights of 1.4 k, 4.3 k, 6.8 k, 13 k, 17 k, 32 k, 49 k, 77 k, 150 k, 350 k, 990 k and 2,600 k Dalton (Fluka, Buchs, Switzerland). The number-average molecular weight (M_n_) and weight-average molecular weight (M_w_) for each HA were estimated.

### 2.5. SOS/umu Test Procedure

The *umu* test was performed in a 96-well round-bottom micro-plate made by polystyrene (Falcon, Franklin Lakes, NJ, USA). The *S. typhimurium* strains were grown in TGA medium (1% Bacto tryptone, 0.5% NaCl and 0.2% glucose) supplemented with 20 μg·mL^−1^ ampicillin at 37 °C for about 12–16 h. The overnight culture was diluted 50-fold with TGA medium and it was incubated at 37 °C until the bacterial density reached absorbance of 0.2 at 595 nm using a common square cell with 10 mm of optical path length. The bacterial culture was subdivided into 200 μL aliquots in the each of the microplate well where by 20 μL of test compound solution and 80 μL of 0.1 M PBS with or without HA and artificial sediment were added to it. The concentration of HA and artificial sediment shown in this manuscript are one in this incubation step in 300 μL of mixture solution. In the case where metabolic activation of test compounds are necessary, 20 μL of test compound, 20 μL of S9-mix (the S9 fractions combined with cofactors) and 60 μL of 0.1 M PBS with or without HA and artificial sediment were added to 200 μL of the bacteria culture. AF-2 and 2-AA were used as positive control chemicals in this study. 2-AA shows metabolically activated to a mutagenic compound by reacting with S9-mix. DMSO was used as negative control. After incubating the mixture for 2 h at 37 °C with gently shaking in incubator (M-BR-024, TAITEC, Tokyo, Japan), the measurements of β-galactosidase activity were performed. The minimal detectable concentration was defined as the concentration that gives twice of signal obtained by control experiment. Prior to the electrochemical measurements, the microplate with the exposed bacteria is kept on ice until usage to prevent bacterial growth.

#### 2.5.1. Spectroscopic Detection Procedure

The spectrophotometric measurements were performed as follows: after incubation at 37 °C for 2 h, the bacterial density at 595 nm (OD_595_) was measured by microplate reader (Model 550, BIO-RAD, Hercules, CA, USA). Ninety μL of the treated bacterial cell suspension were diluted with 120 μL of B-buffer, and then 30 μL ONPG (4.5 mg·mL^−1^ in 0.1 M PBS) were added. The mixture was incubated at 37 °C for 30 min with gentle shaking. The reaction was terminated after 30 min by adding 60 μL of 1 M Na_2_CO_3_. The absorbance was measured at 415 nm (OD_415_) and 548 nm (OD_548_). The units of β-galactosidase activity were calculated based on the formula of Miller [49] as follows: β-galactosidase activity (Unit/A595) = 1,000 × (OD_415_ − 1.75 × OD_548_) / (t × v × OD_595_), where t is the enzymatic reaction time (min) and v is the volume of the bacterial suspension (mL).

#### 2.5.2. Electrochemical Detection Procedure

Hydrodynamic amperometry with a rotating disk electrode (RDE) was done using an electrochemical analyzer (ALS-1200, Bioanalytical Systems Inc. (BAS) (West Lafayette, IN, USA). A glassy carbon disk electrode (6 mm outer diameter, 3 mm inner diameter (BAS)), a silver wire (0.7 mm diameter) and a platinum wire (0.5 mm diameter) were used as working, reference, and counter electrode, respectively. Prior to the measurement, the glassy carbon disk electrode was polished sequentially with 0.3 and 0.05 μm alumina paste and then rinsed well with water.

Forty μL of the *S. typhimurium* solution were sandwiched between the glassy carbon disk electrode surface and a Parafilm^®^ (Pechiney Plastic Packaging Co., Chicago, IL, USA) covered a glass slide as illustrated in Figure 1. The potential was applied to the electrode and the rotation was set at 3,000 rpm. After an equilibration time of 5 s, the current was measured in order to establish a baseline current. At 20 s, 5 μL of 16 mg·mL^−1^ PAPG (final concentration 6.56 mM) were added to the droplet by a micropipette and the current was measured for an additional 20 s. The slope between 30 s and 40 s was corrected by subtracting the background slope, taken between 10 s and 20 s, to give the reaction velocity.

## 3. Results and Discussion

### 3.1. Investigation of Electrochemical Behavior of PAP and PAPG in the Sample with or without S9-mix by Liner Sweep Voltammetry Using RDE System

**Figure 2.**
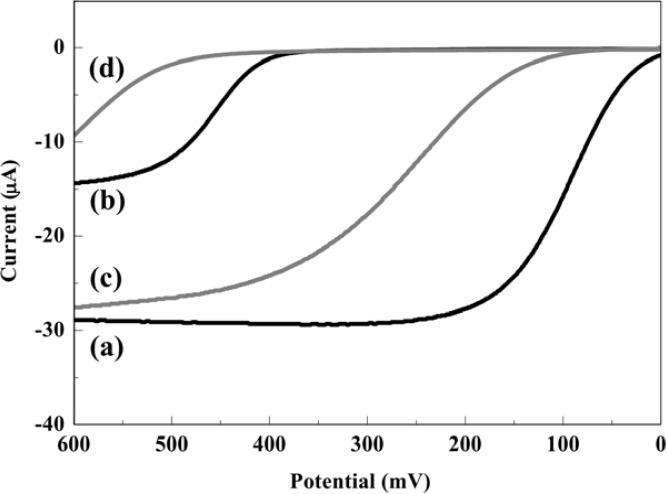
The hydrodynamic liner sweep voltammograms of (**a**) 0.2 mM PAP, (**b**) 0.2 mM PAPG, (**c**) 0.2 mM PAP with S9-mix and (**d**) 0.2 mM PAPG with S9-mix. The measurements were done in a droplet at a scan rate of 100 mV·s^−1^ with a rotation rate of 3,000 rpm.

The electrochemical behaviors of PAP and PAPG at the RDE in 0.1 M PBS with or without S9-mix were investigated by hydrodynamic linear sweep voltammetry as shown in Figure 2. The measurements were performed at a scan rate of 100 mV·s^−1^ with a rotation rate of 3,000 rpm in a 45 μL droplet including 0.2 mM PAP or PAPG with or without 10% of S9-mix. In this assay, the electrochemical oxidation of PAPG should not influence the oxidation of PAP, because PAPG molecules exist at a much higher concentration compared with PAP molecules which are the enzymatic product. In the case without S9-mix, the mass-transfer limited current (Levich current) for PAP and PAPG oxidation was obtained at a potential over 200 mV and 500 mV, respectively. On the other hand, the electrochemical oxidation signals of PAP and PAPG were shifted toward positive potential when S9-mix was added in the sample. However, the mass-transfer limited currents obtained from PAP oxidation were almost the same in the samples with and without S9-mix. These results show that the presence of S9-mix hardly interfered with the electrochemical detection of PAP. Thus, the potential applied in the hydrodynamic choronoamperometric measurements in order to assess the genotoxicity were chosen at 300 mV for the sample without S9-mix and 400 mV for the sample with S9-mix.

### 3.2. Influence of HA and Sediment on the Electrode Response of PAP

**Figure 3.**
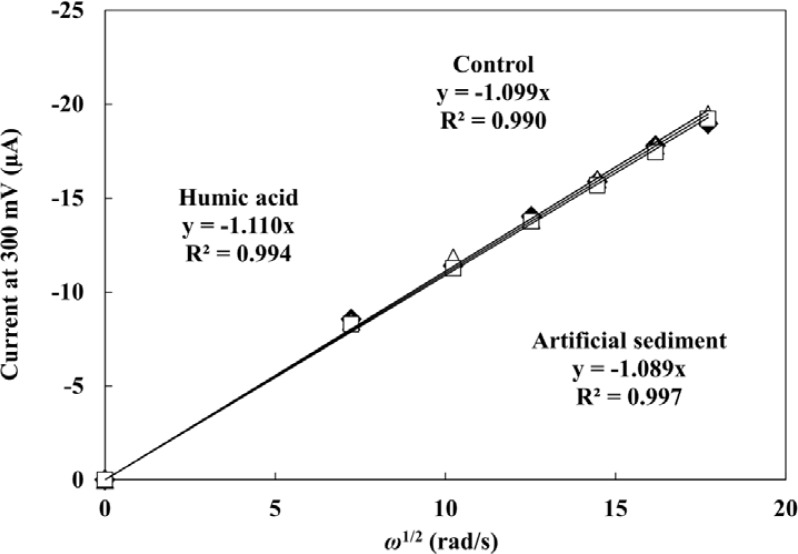
The relationship of the mass-transfer limited current for oxidation of PAP *vs*. square root of rotation angular velocity (*ω*^1/2^). The hydrodynamic liner sweep voltammograms were measured in 0.2 mM PAP droplet (•), including 40 mg·L^−1^ WHA (○), including 1 g·L^−1^ artificial sediment (□) with a different rotation rate of 500, 1,000, 1,500, 2,000, 2,500 and 3,000 rpm at a scan rate of 100 mV·s^−1^.

The influence of HA and sediment present in the sample on the PAP detection were evaluated by hydrodynamic linear sweep voltammetry. Forty five μL of droplet including 0.2 mM PAP were measured with different rotation rates of 500, 1,000, 1,500, 2,000, 2,500 and 3,000 rpm at a scan rate of 100 mV·s^−1^. A completely rigorous hydrodynamic treatment has been given by Levich’s equation [40] as follows: *i*_l_ = 0.620*nFAC*^0^*D*^2/3^*ν*^−1/6^*ω*^1/2^, where *i*_l_ is the mass-transfer limited current, *n* is the number of electrons involved in the reaction, *C*^0^ is the analyte concentration, *ν* is the kinematic viscosity of the fluid, and *ω* is the angular velocity of the disk (2π × rpm). The plots of mass-transfer limited current values against square root of *ω* are shown in Figure 3. The limiting currents showed a good linear relationship with square root of *ω*. This indicates that the electrochemical response of PAP is controlled by the convective mass transport resulting from the hydrodynamic flow caused by RDE. The linear relationship between the limiting currents of PAP and square root of *ω* did not change with the presence of 40 mg·L^−1^ WHA and 1 g·L^−1^ of the artificial sediment. It is clear that the hydrodynamic voltammetric detection for PAP is not affected by the high concentration of DHSs and sediment particles. Therefore, the application of hydrodynamic voltammetric detection to the *umu* test is an effective strategy to achieve the development of a genotoxicity test which can be used for the evaluation of samples including organic matter, suspended solids and soil, *etc*.

### 3.3. Demonstration of Hydrodynamic Electrochemical Genotoxicity Test for AF-2 Using RDE System

**Figure 4.**
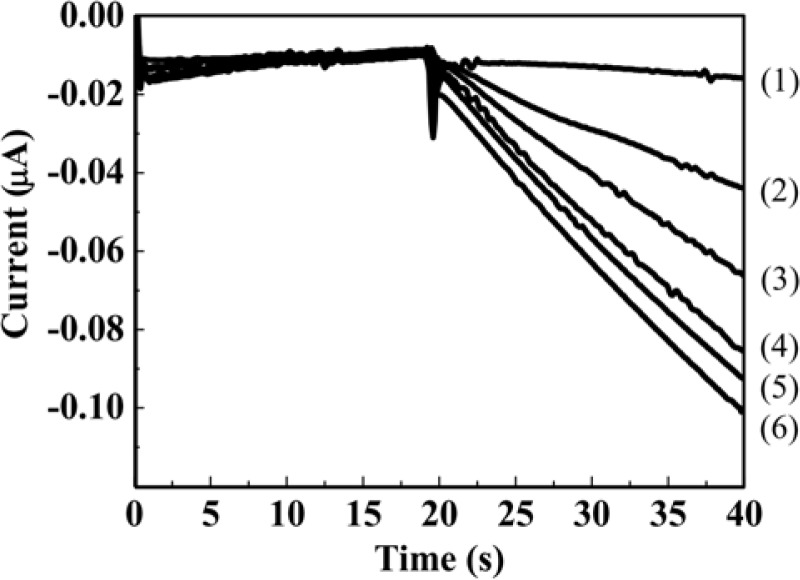
The chronoamperograms for *S. typhimurium* exposed to 30 ng·mL^−1^ AF-2. The measurements were done by the RDE system at 300 mV applied potential with a rotation rate of 3,000 rpm. (**1**) 0 mM, (**2**) 0.82 mM, (**3**) 1.64 mM, (**4**) 3.28 mM, (**5**) 6.56 mM and (**6**) 13.12 mM PAPG was added into the droplet including *S. typhimurium* at 20 s.

The genotoxicity tests based on hydrodynamic chronoamperometry using the RDE system in a microliter droplet were demonstrated for AF-2 as a model genotoxic compound. Figure 4 shows the hydrodynamic chronoamperograms obtained from different concentrations of PAPG as the enzyme substrate. Each concentration of PAPG (5 μL) was added to 40 μL of *S. typhimurium* solution. Prior to the measurements, *S. typhimurium* NM2009 strain was exposed to 30 ng·mL^−1^ AF-2 for 2 h at 37 °C with gentle shaking in an incubator. An obvious increment of anodic current with increasing concentration of PAPG injected into the droplet sample including *S. typhimurium* was obtained within only 40 s.

**Figure 5.**
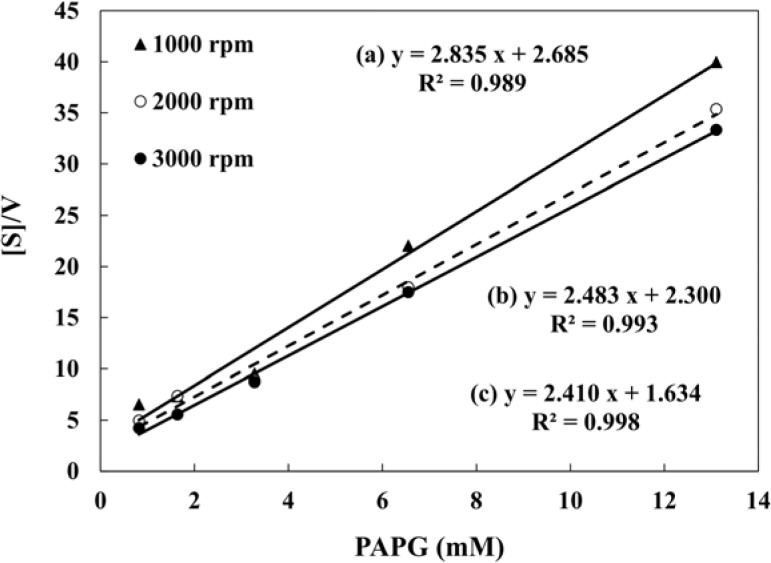
Hanes-Woolf plots of β-galactosidase in *S. typhimurium* which was exposed to 30 ng·mL^−1^ AF-2. The measurements were done by the RDE system at 300 mV applied potential with a rotation rate of 1,000, 2,000 and 3,000 rpm.

The hydrodynamic amperometric responses of *S. typhimurium* with different concentrations of PAPG were fitted according to the Michaelis-Menten equation (*v* = *v*_max_ [PAPG]/(*K*_M_ + [PAPG])). Figure 5 shows the Hanes-Woolf plots of β-galactosidase in *S. typhimurium* obtained by the hydrodynamic voltammetry with a rotation rate of 1,000, 2,000, and 3,000 rpm. The Michaelis-Menten constant (*K*_M_) and the maximum velocity (*v*_max_) obtained from the Hanes-Woolf plots are shown in Table 2. Buchinger *et al*. proposed the *umu* test based on chronoamperometric detection using the screen printed electrode, and the Michaelis-Menten constant was determined to be 1.78 mM [39]. As for this study, the obtained Michaelis-Menten constant was 0.68 mM at the 3,000 rpm. This indicates that the hydrodynamic amperometry with RDE system in a microliter droplet is usable in evaluating enzyme activity from *S. typhimurium* caused by exposure to genotoxins.

### 3.4. Comparison of Genotoxicity Tests with Spectrophotometric and Hydrodynamic Electrochemical Detection

**Figure 6.**
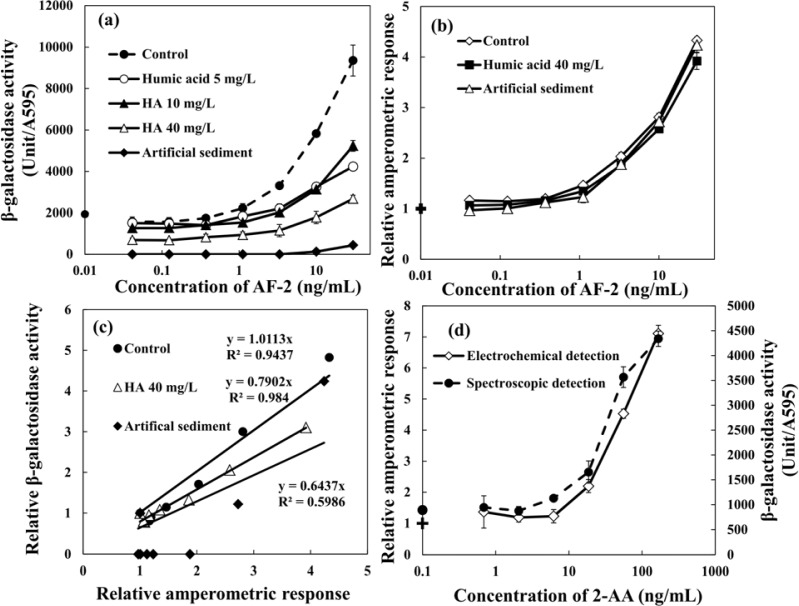
The dose-response curves of AF-2 obtained by the *umu* test based on (**a**) the spectrophotometric detection and (**b**) the hydrodynamic amperometric detection in the sample including 5, 10 and 40 mg·L^−1^ humic acid (WHA) and 1 g·L^−1^ artificial sediment. (**c**) The relationships between the relative amperometric respose and the relative β-galacotosidase acitivity obtained by the control experiment and the conditions in the presence of 40 mg·L^−1^ HA or 1 g·L^−1^ artificial sediment. (**d**) The comparison of dose-response curves of 2-AA obtained by the *umu* test based on spectrophotometric detection and hydrodynamic amperometric detection. Symbol mean + = blank of the electrochemical detection and • = blank of the spectroscopic detection.

The dose-response curves of AF-2 obtained by the regular *umu* test based on spectrophotometric detection are shown in Figure 6(a). As compared with the control experiment, it is clear that the assay was strongly affected by the presence of WHA in the sample. This was even observed at a low concentration of HA (5 mg·L^−1^ of WHA). Moreover, the degree of interference increased with increasing HA concentration and the minimal detectable concentrations for AF-2 increases with increasing concentration of WHA. The presence of 1 g·L^−1^ artificial sediment in the sample strongly affected the genotoxicity assay results, and the genotoxicity of AF-2 was hardly detected in the sample which contained artificial sediment. The relative amperometric responses obtained from the *umu* test based on hydrodynamic amperometric detection using RDE system in a droplet are shown in Figure 6(b). The relative amperometric response represents the rate obtained from sample normalized by the rate of the negative control sample. In contrast with the *umu* test based on spectrophotometric detection, the assay based on hydrodynamic amperometric detection was unaffected by the presence of humic acid and sediment particles. The dose-response curves of AF-2, even in the presence of high concentration of WHA (40 mg·L^−1^) and 1 g·L^−1^ artificial sediment, showed a good correlation with the one obtained from control experiment. Figure 6(c) shows the relationships between the relative amperometric respose and the relative β-galacotosidase acitivity. The results obtained from the control experiment based on these two methods showed good agreement. However, the slope of the relative amperometric respose *vs*. the relative β-galacotosidase acitivity decreased by the presence of 40 mg·L^−1^ WHA and 1 g·L^−1^ artificial sediment. These results indicate that the proposed electrochemical *umu* test can be applied to samples containing HAs and sediment particles, contrary to the common *umu* test based on optical detection that is strongly interfered by the presence of HA and sediment particles.

In addition, the comparison of the *umu* test based on spectrophotometric detection and the hydrodynamic amperometric detection were performed for the metabolically activated genotoxicity of 2-AA as shown in Figure 6(d). Similar dose-response curves were obtained from both methods. Both the spectrophotometric and electrochemical detection methods showed the same value of the estimated minimal detectable concentration for 2-AA. Therefore, this method can also evaluate metabolically activated genotoxicity.

The detection time required for the enzymatic reaction with substrate using this method is only 40 s, which is a good improvement when compared to the spectrophotometric and other electrochemical detections that require much longer times, anywhere from 10 to 60 min [38,39].

### 3.5. The Changes in Genotoxicity of 4-NQO, MX, 1,8-DNP, 1-NP Caused by HAs and Soil Particles

**Figure 7.**
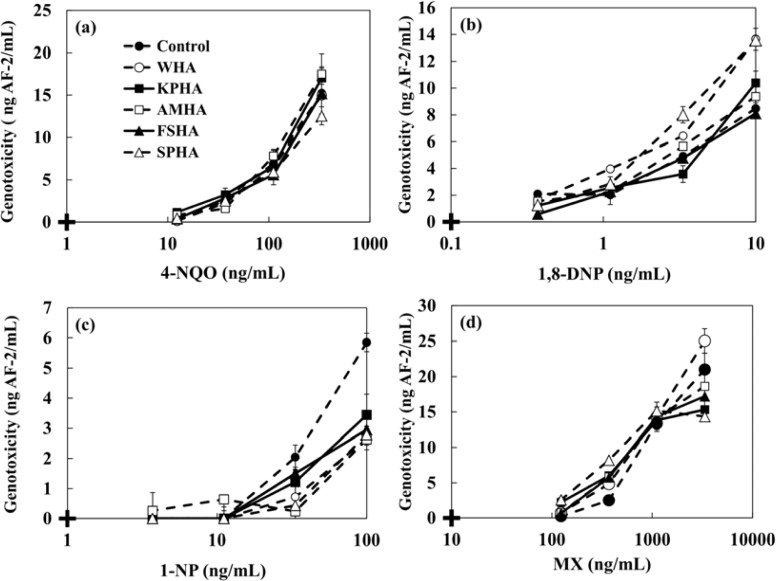
The dose-response curve of (**a**) 4-NQO, (**b**) 1,8-DNP, (**c**) 1-NP and (**d**) MX by the electrochemical detection in the presence of 10 mg·L^−1^ various HAs (WHA, KPHA, AMHA, FSHA, SPHA). Symbol mean + = blank.

The changes in the genotoxicity of 4-NQO, MX, 1,8-DNP and 1-NP caused by the presence of humic acids (WHA, KPHA, AMHA, FSHA, SPHA) and artificial sediment (*Sphagnum* moss peat 4%, kaolin 20%, quartz sand 76%) were evaluated. 4-NQO is known to produce cancer in a number of organs and tissues. MX is a mutagenic and carcinogenic chlorinated franone and it is formed during chlorination of fulvic and humic acid [2,4,6,8]. Many researchers have reported that MX is a disinfection by-product found in drinking water at ng·L^−1^ levels [8,9,11]. 1,8-DNP and 1-NP were used as typical nitroarenes which have been detected in the extracts of diesel and gasoline emission, fly ash particles, cigarette smoke condensates and home heater emissions. Ten mg·L^−1^ HAs or 1 g·L^−1^ artificial sediment were added to each genotoxic compound and left at room temperature in the dark with gentle shaking. After 24 h, the mixture solutions were tested by adding the strain. Table 3 and Figure 7 show the minimal detectable concentrations of 4-NQO, MX, 1,8-DNP and 1-NP and the dose-response curves in the presence of 10 mg·L^−1^ of various HAs. The genotoxicity shown in y-axis of Figure 7 was converted to AF-2 concentration. In the case of 4-NQO, the changes of genotoxicity by the presence of 10 mg·L^−1^ HAs were not observed. An increment of genotoxicity was observed for MX. On the other hand, the presence of HAs mitigated the genotoxicity of 1-NP. The minimal detectable concentrations of each genotoxin in the presence of HAs are shown in Table 3. We investigated a correlation between the genotoxicity changes caused by the presence of HAs and each chemical character of HAs as shown in Table 1. The finding shows that there was no correlation between them.

**Figure 8.**
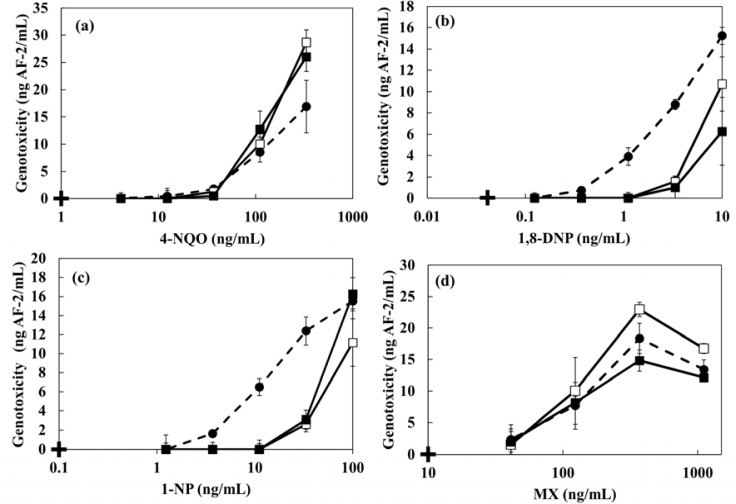
The dose-response curves of (**a**) 4-NQO, (**b**) 1,8-DNP, (**c**) 1-NP and (**d**) MX by the electrochemical detection in the presence of artificial sediment and modified artificial sediment (removed *Sphagnum* moss peat). Symbols mean + = blank of the electrochemical detection, • = control, □ = with artificial sediment, ▪ = with modified artificial sediment.

The dose-response curves and the minimal detectable concentrations of 4-NQO, MX, 1,8-DNP and 1-NP in the presence of 1 g·L^−1^ artificial sediment are shown in Figure 8 and Table 4, respectively. The genotoxicity of 4-NQO and MX did not change by the presence of sediment. However, a decrease of genotoxicity of 1,8-DNP and 1-NP were observed in the sample including sediment. The minimal detectable concentration of 1-NP in the presence of sediment showed 5.8-fold higher increase than the case without artificial sediment (Table 4). The reduction of genotoxicity could be caused by the changed bioavalability because hydrohobic genotoxic compounds with a high hydrophobic property tend to adsorb onto sendiment particles. The log *K*_ow_ values of 1,8-DNP and 1-NP are 4.57 and 4.75, respectively. Zang *et al*. have also been reported that genotoxicity of pesticides against the earthworm (*Eisenia fetida*) was changed by the presence of artificial sediment [37]. In addition, similar results were obtained in the case of modified artificial sediment in which organic components were removed. These results indicate that the genotoxicity of hydrophobic organic compounds is influenced by the presence of inorganic constituents in sediments more than by organic components. Sun *et al*. reported that silica particles (75 μm of particle size and 6 nm of pore size) showed a high sorption constant (7.60) for both naphthalene and pyrene [23]. The particle size of the kaolin and quartz sand that comprise the artificial sediment used in this study are ≤25 μm. Therefore, it seems that the adhesion of genotoxins with a high hydrophobicity to the modified artificial sediment greatly affected the genotoxicity.

## 4. Conclusions

A new *umu* test based on hydrodynamic electrochemical detection in a microliter droplet using the rotating disk electrode (RDE) was developed. The dose-response curves of AF-2 obtained from the *umu* test based on spectrophotometric and electrochemical detection were compared. It is obvious from the comparison that even though the assay based on spectrophotometric detection is strongly influenced by the presence of humic acid (HA) and artificial sediment, the proposed assay is still able to evaluate the genotoxicity of AF-2 in the presence of 40 mg·L^−1^ HA and 1 g·L^−1^ artificial sediment. The analysis time for incubation step with NM2009 strain and genotoxins is about 2 h. One of the main advantages of this method is shorter detection time for enzymatic reaction with substrate (40 s) when compared to spectrophotometric and other electrochemical detection methods proposed in previous assays which require much longer times of 10 to 60 min. The changes in the genotoxicity of AF-2, 4-NQO, MX, 1,8-DNP and 1-NP due to the presence of HAs and artificial sediment were also investigated. The observations showed that the minimal detectable concentration of 1-NP increased by the presence of 10 mg·L^−1^ HAs. Fourthermore, the results also indicated that the presence of 1 g·L^−1^ sediment strongly mitigated the genotoxicity of chemicals with high hydrophobic properties, *i.e.*, a high log *K*_ow_ value, such as 1,8-DNP (4.57) and 1-NP (4.75). There wwas no such reduction in the genotoxicity for AF-2 (1.14), 4-NQO (0.82) and MX (0.61), which are also genotoxic compounds, but with a lower log *K*_ow_. The proposed hydrodynamic electrochemical method can be used in assessing the genotoxicity of real sediments, soils and suspended solids.

## Figures and Tables

**Table 1.** The results for characterization of HAs (WHA, AMHA, KPHA, SPHA, FSHA).

**Sample Name**	***E*280^a^**	***E*400^b^**	***E*4/*E*6^c^**	***E*2/*E3*^d^**	**Total acidity (meq·g^−1^)**	**Carboxylic acid (meq·g^−1^)**	**Phenolic hydroxyl groups (meq·g^−1^)^e^**
WHA	91.6	33.9	4.20	2.28	5.24	2.08	3.16
AMHA	43.9	13.4	4.38	3.12	9.82	4.38	4.36
KPHA	59.5	19.3	6.38	2.58	13.13	3.74	9.39
SPHA	43.5	11.8	7.75	3.03	5.09	4.02	1.07
FSHA	38.6	10.6	5.40	3.10	6.96	3.73	4.74

**Sample Name**	**M_n_ (Da)^f^**	**M_w_ (Da)^g^**	**M_w_/M_n_**	**%C**	**%H**	**%N**	**%O**	**%S**	**%Ash**
WHA	490	1,548	3.46	63.2	3.3	1.3	31.3	0.9	0.1
AMHA	1,604	11,852	7.39	52.2	5.0	3.8	38.3	0.4	0.3
KPHA	1,117	3,812	3.41	52.9	4.4	1.9	39.6	0.3	1.0
SPHA	1,869	18,541	9.92	52.7	5.1	2.5	37.4	0.6	1.7
FSHA	2,027	14,084	6.95	51.1	5.2	4.0	37.7	0.4	1.1

Absorptivities at

a 280 nm and

b 400 nm (L·cm^−1^·g-C^−1^).

c Ratio of absorbance at 400 and 600 nm.

d Ratio of absorbance at 250 and 365 nm.

e The contents of phenolic hydroxyl groups were calculated by subtracting the carboxylic acid contents from the total acidity.

f Number-average molecular weight (M_n_).

g Weight-average molecular weight (M_w_).

**Table 2.** *V*_max_ and Michaelis constant (*K*_M_) in the case of the electrode rotated from 1,000–3,000 rpm.

**Rotation rate (rpm)**	***V*_max_**	***K*_M_ (mM)**
1,000	0.35	0.95
2,000	0.40	0.93
3,000	0.42	0.68

**Table 3.** The minimal detectable concentration of 4-NQO, 1,8-DNP, 1-NP, MX by the electrochemical detection in the case of without and with 10 mg·L^−1^ various humic acids (WHA, KPHA, AMHA, FSHA, SPHA). The values are mean (n = 3).

**Minimal detectable concentration (ng·mL^−1^)**				
	4-NQO	1,8-DNP	1-NP	MX
Control	44.3 ± 6.6	2.0 ± 0.9	44.6 ± 5.1	219.8 ± 15.7
WHA	50.9 ± 11.0	1.2 ± 0.4	76.8 ± 2.9	167.7 ± 35.9
KPHA	41.1 ± 8.9	2.1 ± 0.5	55.1 ± 10.1	111.6 ± 32.4
AMHA	43.8 ± 7.0	1.1 ± 0.1	80.2 ± 16.7	156.7 ± 45.7
FSHA	52.4 ± 13.8	1.5 ± 0.2	101.6 ± 9.2	159.7 ± 55.6
SPHA	49.0 ± 7.9	0.9 ± 0.2	87.7 ± 15.3	80.1 ± 22.8

**Table 4.** The minimal detectable concentration of 4-NQO, 1,8-DNP, 1-NP, MX by the electrochemical detection in the presence of 1 g·L^−1^ artificial sediment or 1 g·L^−1^ modified artificial sediment (removed *Sphagnum* moss peat). The values are mean (n = 3).

**Sample**	**Minimal detectable concentration (ng·mL^−1^)**			
	**4-NQO**	**1,8-DNP**	**1-NP**	**MX**
Control	39.3 ± 4.8	1.0 ± 0.1	5.6 ± 0 .9	46.8 ± 5.9
Artificial sediment	46.9 ± 3.5	4.2 ± 0.3	32.5 ± 3.2	51.7 ± 3.6
Modified artificial sediment	39.9 ± 2.7	6.2 ± 0.5	36.2 ± 4.0	50.0 ± 4.8
